# Supplementary material for: Psychological Impacts and Post-Traumatic Stress Disorder among People under COVID-19 Quarantine and Isolation: A Global Survey
Source: Int J Environ Res Public Health. 2021 May 26;18(11):5719. doi: 10.3390/ijerph18115719 (PMC8199241; doi:10.3390/ijerph18115719)
Supplement: Supplementary file 1 [file ijerph-18-05719-s001.zip › ijerph-1180873-supplementary/supp/IESR-Suppleemtary-file-S1.pdf]

Supplementary file S1

***Members of TMGH-Global COVID-19 Collaborative* (List of global collaborators)**

Collaborative members (name, affiliation, ORCID ID) and their contributions are listed below.

Writing Team (protocol development, project management, data analysis, interpretation, writing, review/ revisions)

TMGH-Global COVID-19 Collaborative: Nguyen Tran Minh Duc (Faculty of Medicine, University of Medicine and Pharmacy at Ho Chi Minh City, Ho Chi Minh City, Vietnam); Shamael Thabit Mohammed Alhady (Faculty of Medicine, University of Gezira, Wad Medani, Sudan); Luu Ngoc Mai (Department of Internal Medicine, University of Medicine and Pharmacy at Ho Chi Minh City, Ho Chi Minh City, Vietnam); Shyam Prakash Dumre (Central Department of Microbiology, Tribhuvan University, Kathmandu, Nepal); Amr K. Hassan (Faculty of Medicine, South Valley University, Qena, Egypt); Tran Van Giang (Department of Infectious Disease, Hanoi Medical University, Hanoi, Vietnam); Le Van Truong (Traditional Medicine Hospital, Ministry of the Public Security, Hanoi, Vietnam); Rohanti Ravikulan (Southern Adelaide Local Health Network, Adelaide, Australia); Akshay Raut (Rajarshee Chhatrapati Shahu Maharaj Govt Medical College, Kolhapur, India); Farouq Muhammad Dayyab (Infectious Disease Hospital, Kano, Nigeria); Vu Thi Thu Trang (National Hospital of Traditional Medicine, Hanoi, Vietnam); Le Quang Loc (Faculty of Medicine, University of Medicine and Pharmacy at Ho Chi Minh City, Ho Chi Minh City, Vietnam); Pham Ngoc Thach (Department of Infectious Disease, Hanoi Medical University, Hanoi, Vietnam); Nguyen Tien Huy (School of Tropical Medicine and Global Health, Nagasaki University, Nagasaki, Japan).

E-mails and ORCID:

NTMD: minhduc1298@gmail.com; 0000-0002-9333-7539

STMA: shamaelthabit4321@gmail.com; 0000-0001-7463-3300

LMN: ngmai3288@gmail.com; 0000-0002-8129-1764

AKH: dramrhassan@yahoo.com ; 0000-0002-8281-9268

TVG: giangminh08@gmail.com; 0000-0002-3781-2331

LVT: bs.vantruong@gmail.com; 0000-0002-7474-0453

RR: r.rohanti@gmail.com; 0000-0002-6402-1279

SPD: sp.dumre@gmail.com; 0000-0002-4072-0745

AR: akshayraut2610@gmail.com; 0000-0002-0210-989X

FMD: farouqmuhd@yahoo.com; 0000-0001-8920-6483

VTTT: dr.vutrang@gmail.com; 0000-0001-7152-4299

LQL: lequangloc.md@gmail.com; 0000-0001-6363-9862

PNT: phamngocthachnhtd@gmail.com

NTH: tienhuy@nagasaki-u.ac.jp; 0000-0002-9543-9440

Collaborators (obtaining ethical approval as and when needed at local site, study participant recruitment at local site and data collection)

Kamal Ranabhat (Ministry of Health and Population, Kathmandu, Nepal; Institute of Medicine, Tribhuvan University, Kathmandu, Nepal); Nahida Al Habaj (Faculty of medicine, University of Tours, France); Salma Elnoamany (Faculty of Medicine, Menoufia University, Menoufia, Egypt); Jeza Muhamad Abdul Aziz (Medical Laboratory Science, College health science, University of Human Development,

Sulaymaniyah, Kurdistan region, Iraq; Baxshin Research Center, Baxshin Hospital, Sulaymaniyah, Kurdistan Region, Iraq); Kevin Thurston Crispino (Health Emergency Management Bureau, Department of Health, Manila, Philippines); Suhir Alsuwiyah (Faculty of Medicine, University of Tripoli, Libya); Rangin Muhamad Hussein (Baxshin Research Center, Baxshin Hospital, Sulaymaniyah, Kurdistan region, Iraq); Dashne Jalal Hama (Baxshin Research Center, Baxshin Hospital, Sulaymaniyah, Kurdistan region-Iraq); Graca Jaqueline Vanessa Morena (Faculty of Medicine, Trisakti University, Jakarta, Indonesia & America Evangelical University, Los Angeles CA, USA); Ian Christopher Naungayan Rocha (School of Medicine, Centro Escolar University, Manila, Philippines); Dmytro Pavlenko (Department of Ophthalmology, Bogomolets National Medical University, Kyiv, Ukraine); Hemin Fatih Hama Kareem (Department of Psychiatry, Mental Health Center, Baxshin Hospital Sulaymaniyah, Kurdistan region, Iraq); Rifath Jahan Antora (Kunming Medical University, Kunming, Yunnan, China); Andrés Sebastián Estrella Lopez (Faculty of Medicine, Pontificia Universidad Católica del Ecuador, Ecuador); Mohammed Ibrahim Mohialdeen Gubari (Department of Family and Community Medicine, College of Medicine, University of Sulaimaniyah, Sulaimaniyah, Kurdistan Region, Iraq); Nut Koonrungsesomboon (Department of Pharmacology, Faculty of Medicine, Chiang Mai University, Chiang Mai, Thailand); Chidchanok Ruengorn (Department of Pharmaceutical Care, Faculty of Pharmacy, Chiang Mai University, Chiang Mai, Thailand); Surapon Nochaiwong (Department of Pharmaceutical Care, Faculty of Pharmacy, Chiang Mai University, Chiang Mai,

Thailand); Penkarn Kanjanarat (Department of Pharmaceutical Care, Faculty of Pharmacy, Chiang Mai University, Chiang Mai, Thailand); Mingkwan Na Takuathung (Department of Pharmacology, Faculty of Medicine, Chiang Mai University, Chiang Mai, Thailand); Samah M.elhassan (Alneelain University, Faculty of Medicine, Khartoum, Sudan); Ali Dzhemiliev (Shalimov National Institute of Surgery and Transplantology, Kyiv, Ukraine); Brianda del Pilar Gómez Olvera (Autonomous University of Mexico State, Mexico); Md Ariful Haque (Department of Orthopedic Surgery, Yan an hospital affiliated to kunming medical university, Kunming, Yunnan, China); Irfan Ullah (Kabir Medical College, Gandhara University, Peshawar, Pakistan); Irida Dajti (UHOG 'Koço Gliozheni', Albania); Venkatesh U (Department of Community Medicine, Vardhman Mahavir Medical College & Safdarjung Hospital, New Delhi, India); Zair Hassan (Cardiology department, Lady Reading Hospital, Peshawar, Pakistan); Zakarya Salem Ahmed (Faculty of Medicine, Cairo University, Egypt); Mawada fath Alrahman (University of Bakhat alruda, Sudan); Ton That Khanh (Faculty of Medicine, School of Medicine and Pharmacy, Da Nang University, Da Nang City, Vietnam); Ahmad Taysir Atieh Qarawi (Lower Westchester Medical Associates, P.C., Mount Vernon, NY 10550, USA); Iryna Kudlatska-Tyshko (Shupyk National Medical Academy of Postgraduate Education, Kyiv, Ukraine); Doaa Mahmoud Eisa Sabir (Faculty of Medicine, University of Gezira, Wad Medani, Sudan); Iftikhar Ali (Paraplegic Center, Hayatabad Peshawar, Pakistan); Sze Jia Ng (School of Medical Sciences, Universiti Sains Malaysia, Kelantan, Malaysia); Pham Trung Nghia (Faculty of Medicine, Pham

Ngoc Thach University of Medicine, Ho Chi Minh City, Vietnam); Mariia Pavlenko (Kyiv City Pediatric Diagnostic Center, Kyiv, Ukraine); Kirellos Said Abbas (Faculty of Medicine, Alexandria University, Alexandria, Egypt); Fatma A. Monib (Faculty of Medicine, Assiut University, Assiut, Egypt); Aliaa Effat Said (Faculty of Medicine, Assiut University, Assiut, Egypt); Shehab Fathy Ahmed (Faculty of Medicine, Assiut University, Assiut, Egypt); Ziad Hassan Hamed (Faculty of Medicine, Assiut University, Assiut, Egypt); Mariam Albatoul Nageh (Faculty of Medicine, Assiut University, Assiut, Egypt); Esraa Sayed (Faculty of Medicine, Assiut University, Assiut, Egypt); Mosa Shibani (Faculty of Medicine, Syrian Private University, Damascus, Syrian Arab Republic); Hlma Ismail (Faculty of Medicine, Syrian Private University, Damascus, Syrian Arab Republic); Mhd Amin Alzabibi (Faculty of Medicine, Syrian Private University, Damascus, Syrian Arab Republic); Bisher Sawaf (Faculty of Medicine, Syrian Private University, Damascus, Syrian Arab Republic); Hoda Aly Mohamed Omran (Faculty of Medicine, Alexandria University, Alexandria, Egypt); Chiristine Samuel Rezq (Faculty of Medicine, Alexandria University, Alexandria, Egypt); mohamed ibrahim abdo ibrahim (Faculty of Medicine, Alexandria University, Alexandria, Egypt); Marina Samy Ragheb (Faculty of Medicine, Alexandria University, Alexandria, Egypt); Jola Kërpaçi (UHOG 'Koço Gliozheni', Albania); Enxhi Vrapi (UHOG 'Koço Gliozheni', Albania); Juwie Chuah (School of Medical Sciences, Universiti Sains Malaysia, Kelantan, Malaysia); Yi Liang Lim (School of Medical Sciences, Universiti Sains Malaysia, Kelantan, Malaysia); Yap Siang Jee (School of Medical Sciences, Universiti Sains Malaysia,

Kelantan, Malaysia); Li Chuin Chong (School of Data Sciences, Perdana University, Malaysia); Boughalem Younes (Faculty of Medicine and Pharmacy, Cadi Ayyad University, Marrakesh, Morocco); Ahmed Hisham Mohamed Hamed (Faculty of Medicine, Cairo University, Cairo, Egypt); Adriana Viola Miranda (Faculty of Medicine, University of Indonesia, Jakarta, Indonesia); Rachel Silency Aritonang (Faculty of Medicine, Trisakti University, Jakarta, Indonesia; Yadika General Hospital, Indonesia); Atsuko Imoto (School of Tropical Medicine and Global Health, Nagasaki University, Japan), Kazumi Kubota (Department of Biostatistics, Yokohama City University, Japan), Koji Aoki (Graduate School of Nursing, Chiba University, Japan); Pradip Gyanwali (Nepal Health Research Council, Kathmandu, Nepal); Meghnath Dhimal (Nepal Health Research Council, Kathmandu, Nepal); Renu Bhandari Dumre (School of Tropical Medicine and Global Health, Nagasaki University, Nagasaki, Japan); Guna Nidhi Sharma (Ministry of Health and Population, Kathmandu, Nepal); Pallavi Koirala (Nepal Health Research Council, Kathmandu, Nepal); Kriti Adhikari (Nepal Health Research Council, Kathmandu, Nepal); Filipa Lucas (Faculdade de Medicina da Universidade de Lisboa); Joyce Nicole Pineda Ordóñez (Faculty of Medicine, Catholic University of Honduras, Honduras); José Tomás Ordóñez Aburto (Faculty of Medicine, Universidad de La Frontera, Temuco, Chile); Roman Pavlenko (Bogomolets National Medical University, Kyiv, Ukraine); Somia Iqtadar (King Edward Medical University Pakistan); Usman Ghani (Directorate General Health Services Punjab); Sami Ullah Mumtaz (North Medical Ward, KEMU/Mayo Hospital, Lahore, Pakistan)

Email and ORCID

SPD: sp.dumre@gmail.com; 0000-0002-4072-0745

KR: kamal_raj7@iom.edu.np; 0000-0002-3396-7440

NAH: nahidaalhabaj@hotmail.com; 0000-0002-2275-831X

SE: Salmaalnomany6@gmail.com; 0000-0002-6415-9948

JMA: jeza1981@gmail.com; 0000-0002-1522-1880

KTC: kevincrispino16@gmail.com; 0000-0002-6505-2982

SA: suhir.alhadi@gmail.com; 0000-0003-1059-4431

RMH: rangen991@gmail.com; 0000-0001-5938-2224

DJH: ddashnee@gmail.com; 0000-0002-6008-8222

GJVM: moresoares11@gmail.com; 0000-0002-3531-0370

ICNR: giannisrocha@gmail.com; 0000-0002-8775-6876

DP: pavlenkomd@gmail.com; 0000-0001-8761-9559

HFHK: dr.hemin@gmail.com; 0000-0002-8119-5246

RJA: dr.rifathjahan060@gmail.com; 0000-0003-0848-9456

ASEL: andresestrella823@gmail.com; 0000-0003-1929-7548

MIMG: mohammed.jubari@gmail.com; 0000-0002-2873-3981

NK: nkoonrung@gmail.com; 0000-0003-4649-597X

CR: chidchanok.r@elearning.cmu.ac.th; 0000-0001-7927-1425

SN: surapon.nochaiwong@gmail.com; 0000-0003-1100-7171

PK: penkarnk@hotmail.com; 0000-0002-8160-5444

MN: k_mingkwan@hotmail.com; 0000-0003-4240-5367

SME: Samahm.elhassan@gmail.com; 0000-0001-8367-9036

AD: alijemilev@gmail.com; 0000-0002-0529-7902

BPGO: gom-olv@live.com.mx; 0000-0002-5905-8616

MAH: arifulhaque58@gmail.com; 0000-0003-4632-5153

IU: irfanullahecp2@gmail.com; 0000-0003-1100-101X

ID: irida.dajti@gmail.com; 0000-0002-8449-6829

VU: venkatesh2007mbbs@gmail.com; 0000-0001-8059-9364

ZH: zair.hassan7272@gmail.com; 0000-0001-9789-0270

ZSA: zakarya.a7med@gmail.com

MFA: mawadafath74@gmail.com; 0000-0003-4721-8536

TTK: khanhtonthat08@gmail.com; 0000-0003-3462-4244

ATAQ: ahmadtqarawi@gmail.com; 0000-0001-7278-9889

IKT: irynakudlatska@gmail.com; 0000-0002-2700-1455

DMES: doaaalesawy17@gmail.com; 0000-0002-2664-9559

IA: Iftikharalijan@gmail.com; 0000-0002-0809-7586

SJN: elaine-1_ng@outlook.com; 0000-0001-5353-6499

PTN: phtrungnghia@gmail.com; 0000-0003-4790-9687

MP: kukushkinamd@gmail.com; 0000-0002-9564-6730

KSA: kirellossaid98@gmail.com; 0000-0003-0339-9339

FA: fatma.ahmed931977@gmail.com; 0000-0001-8456-1603

AES: aliaaeffat30@gmail.com; 0000-0002-3828-6038

SFA: shehabfathy134@gmail.com; 0000-0002-6964-247X

ZHH: zetairon@gmail.com; 0000-0001-6587-8517

MAN: mariam.16266094@med.aun.edu.eg; 0000-0002-7937-4229

ES: esraajamaleldeeb@gmail.com; 0000-0003-0290-5228

MS: moosa.shibani@gmail.com; 0000-0002-4424-2728

HI: Helmaismail997@gmail.com; 0000-0003-4329-9519

MAA: ameenzabibi@gmail.com; 0000-0003-4050-3051

BS: bishersawaf.94@gmail.com; 0000-0001-6751-619X

HAMO: hodaomran100@outlook.com; 0000-0003-1840-3599

CSR: chiristinesamuel@gmail.com; 0000-0002-7040-4268

MIAI: mohamedibrihem2012@gmail.com; 0000-0002-5216-7545

MSR: marinasamy25@gmail.com; 0000-0002-7691-2562

JK: jolamedicine16@gmail.com; 0000-0002-6893-477X

EV: enxhivrapi@gmail.com; 0000-0002-2767-5599

JC: juwiechuah@live.com; 0000-0001-6992-3901

YLL: lim.95@hotmail.com; 0000-0001-6152-1959

YSJ: jeeys@hotmail.my

LCC: lichuinchong@gmail.com; 0000-0002-3574-1365

BY: nsbm2000@gmail.com; 0000-0001-6903-5146

AHMH: ahmed.hamed@students.kasralainy.edu.eg; 0000-0002-0843-532X

AVM: adrianaviola99@gmail.com; 0000-0001-8548-1592

RSA: aritonangrachel@gmail.com; 0000-0003-2702-2691

AI: imoto@nagasaki-u.ac.jp

DKK: kubotaa@yokohama-cu.ac.jp

KA: koji-aoki@chiba-u.jp

PG: prgyawali@nhrc.gov.np;n0000-0001-8609-6717

MD: meghdhimal2@gmail.com; 0000-0001-7176-7821

RBD: renu.bdumre@gmail.com; 0000-0001-8039-0309

GNS: drgunish@gmail.com; 0000-0003-2278-3803

PK: pallavik23@gmail.com

KA: adhkriti@gmail.com

FL: Filipa-lucas@campus.ul.pt; 0000-0001-7651-7194

JNPO: jn.pineda94@gmail.com; 0000-0002-0953-8742

JTOA: jtordonezaburto@gmail.com; 0000-0001-8705-4041

RP: tjroma1@gmail.com; 0000-0001-8070-5649

SI: Somia.iqtadar@gmail.com; 0000-0002-8482-9355

UG: usman293eb@gmail.com

SUM: drsumumtaz@gmail.com

TRANSLATION TEAMS

Albanian

Irida Dajti (UHOG 'Koço Gliozheni', Tirana, Albania), Enxhi Vrapi (UHOG 'Koço Gliozheni', Tirana, Albania), Jola Kërpaçi (UHOG 'Koço Gliozheni', Tirana, Albania)

Arabic

Kirellos Said Abbas (Faculty of Medicine, Alexandria University, Alexandria, Egypt), Fatma A. Monib (Faculty of Medicine, Assiut University, Assiut, Egypt), Aliaa Effat Said (Faculty of Medicine, Assiut University, Assiut, Egypt), Shehab Fathy Ahmed (Faculty of Medicine, Assiut University, Assiut, Egypt), Ziad Hassan Hamed (Faculty of Medicine, Assiut University, Assiut, Egypt), Mariam Albatoul Nageh (Faculty of Medicine, Assiut University, Assiut, Egypt), Esraa Sayed (Faculty of Medicine, Assiut University, Assiut, Egypt), Mosa Shibani (Faculty of Medicine, Syrian Private University, Damascus, Syrian Arab Republic), Hlma Ismail (Faculty of Medicine, Syrian Private University, Damascus, Syrian Arab Republic), Mhd Amin Alzabibi (Faculty of Medicine, Syrian Private University, Damascus, Syrian Arab Republic), Bisher Sawaf (Faculty of Medicine, Syrian Private University, Damascus, Syrian Arab Republic), Hoda Aly Mohamed Omran (Faculty of Medicine, Alexandria University, Alexandria, Egypt), Chiristine Samuel Rezq (Faculty of Medicine, Alexandria University, Alexandria, Egypt), Mohamed Ibrahim Abdo Ibrahim (Faculty of Medicine, Alexandria University, Alexandria, Egypt), Marina Samy Ragheb (Faculty of Medicine, Alexandria University, Alexandria, Egypt)

Bengali

Md Ariful Haque (Department of Orthopedic Surgery, Yan an hospital affiliated to Kunming Medical University, Kunming, Yunnan, China), Rifath Jahan Antora (Kunming Medical University, Kunming, Yunnan, China)

Chinese

Sze Jia Ng (School of Medical Sciences, Universiti Sains Malaysia, Kelantan, Malaysia), Juwie Chuah (School of Medical Sciences, Universiti Sains Malaysia, Kelantan, Malaysia), Yi Liang Lim (School of Medical Sciences, Universiti Sains Malaysia, Kelantan, Malaysia), Yap Siang Jee (School of Medical Sciences, Universiti Sains Malaysia, Kelantan, Malaysia), Li Chuin Chong (School of Data Sciences, Perdana University, Serdang, Malaysia)

Filipino

Ian Christopher Naungayan Rocha (School of Medicine, Centro Escolar University, Manila, Philippines), Kevin Thurston Crispino (Health Emergency Management Bureau, Department of Health, Manila, Philippines)

French

Boughalem Younes (Faculty of Medicine and Pharmacy, Cadi Ayyad University, Marrakesh, Morocco)

German

Ahmed Hisham Mohamed Hamed (Faculty of Medicine, Cairo University, Cairo, Egypt)

Hindi

Venkatesh U (Department of Community Medicine, Vardhman Mahavir Medical College & Safdarjung Hospital, New Delhi, India)

Indonesian

Graca Jaqueline Vanessa Morena (Faculty of Medicine, Trisakti University - Jakarta, Indonesia; America Evangelical University, Los Angeles CA, USA), Adriana Viola

Miranda (Faculty of Medicine, University of Indonesia, Jakarta, Indonesia), Rachel Silency Aritonang (Faculty of Medicine, Trisakti University, Jakarta, Indonesia; Yadika General Hospital, Jakarta, Indonesia)

Japanese

Atsuko Imoto (School of Tropical Medicine and Global Health, Nagasaki University, Nagasaki, Japan), Kazumi Kubota (Department of Biostatistics, Yokohama City University, Yokohama, Japan), Koji Aoki (Graduate School of Nursing, Chiba University, Chiba, Japan)

Korean

Ton That Khanh (Faculty of Medicine, School of Medicine and Pharmacy, Da Nang University, Da Nang City, Vietnam)

Kurdish

Jeza Muhamad Abdul Aziz (Medical Laboratory Science, College health science, University of Human Development, Sulaymaniyah, Kurdistan region, Iraq; Baxshin Research Center, Baxshin Hospital, Sulaymaniyah, Kurdistan Region; Iraq), Mohammed Ibrahim Mohialdeen Gubari (Department of Family and Community Medicine, College of Medicine, University of Sulaimaniyah, Sulaimaniyah, Kurdistan Region, Iraq), Hemin Fatih Hama Kareem (Department of Psychiatry, Mental Health Center, Baxshin Hospital Sulaymaniyah, Kurdistan region, Iraq), Rangin Muhamad Hussein (Baxshin Research Center, Baxshin Hospital, SulaymanKelantaniyah-Kurdistan region, Iraq)

Malay

Sze Jia Ng (School of Medical Sciences, Universiti Sains Malaysia, Malaysia), Juwie Chuah (School of Medical Sciences, Universiti Sains Malaysia, Kelantan, Malaysia), Yi Liang Lim (School of Medical Sciences, Universiti Sains Malaysia, Kelantan, Malaysia), Ahis Siang Jee (School of Medical Sciences, Universiti Sains Malaysia, Kelantan, Malaysia), Li Chuin Chong (School of Medical Sciences, Universiti Sains Malaysia, Kelantan, Malaysia)

Malayalam

Venkatesh U (Department of Community Medicine, Vardhman Mahavir Medical College & Safdarjung Hospital, New Delhi, India)

Nepali

Shyam Prakash Dumre (Central Department of Microbiology, Tribhuvan University, Kathmandu, Nepal; Institute of Tropical Medicine, Nagasaki University, Japan), Kamal Ranabhat (Ministry of Health and Population, Kathmandu, Nepal), Renu Bhandari Dumre (School of Tropical Medicine and Global Health, Nagasaki University, Nagasaki, Japan), Pallavi Koirala (Nepal Health Research Council, Kathmandu, Nepal), Kriti Adhikari (Nepal Health Research Council, Kathmandu, Nepal)

Pastho

Irfan Ullah (Kabir Medical College, Gandhara University, Peshawar, Pakistan)

Portuguese

Filipa Lucas (Faculty of Medicine, University of Lisbon, Lisbon, Portugal)

Russian

Mariia Pavlenko (Kyiv City Pediatric Diagnostic Center, Kyiv, Ukraine), Dmytro Pavlenko (Department of Ophthalmology, Bogomolets National Medical University, Kyiv, Ukraine)

Spanish

Ahmad Taysir Atieh Qarawi (Lower Westchester Medical Associates, P.C., Mount Vernon, NY 10550, USA), Joyce Nicole Pineda Ordóñez (Faculty of Medicine, Catholic University of Honduras, Honduras), José Tomás Ordóñez Aburto (Faculty of Medicine, Universidad de La Frontera, Temuco, Chile), Andrés Sebastián Estrella Lopez (Faculty of Medicine, Pontificia Universidad Católica del Ecuador, Ecuador), Brianda del Pilar Gómez Olvera (Autonomous University of Mexico State, Mexico)

Tamil

Venkatesh U (Department of Community Medicine, Vardhman Mahavir Medical College & Safdarjung Hospital, New Delhi, India)

Thai

Nut Koonrungsesomboon (Department of Pharmacology, Faculty of Medicine, Chiang Mai University, Chiang Mai, Thailand), Chidchanok Ruengorn (Department of Pharmaceutical Care, Faculty of Pharmacy, Chiang Mai University, Chiang Mai, Thailand), Surapon Nochaiwong (Department of Pharmaceutical Care, Faculty of Pharmacy, Chiang Mai University, Chiang Mai, Thailand), Penkarn Kanjanarat (Department of Pharmaceutical Care, Faculty of Pharmacy, Chiang Mai University, Chiang Mai, Thailand), Mingkwan Na Takuathung (Department of Pharmacology, Faculty of Medicine, Chiang Mai University, Chiang Mai, Thailand)

Ukrainian

Dmytro Pavlenko (Department of Ophthalmology, Bogomolets National Medical University, Kyiv, Ukraine), Mariia Pavlenko (Kyiv City Pediatric Diagnostic Center, Kyiv, Ukraine), Roman Pavlenko (Bogomolets National Medical University, Kyiv, Ukraine), Iryna Kudlatska-Tyshko (Shupyk National Medical Academy of Postgraduate Education, Kyiv, Ukraine)

Urdu

Somia Iqtadar (King Edward Medical University, Lahore, Pakistan), Usman Ghani (Directorate General Health Services Punjab, Lahore, Pakistan), Sami Ullah Mumtaz (North Medical Ward, KEMU/Mayo Hospital, Lahore, Pakistan), Asad Ali Khan (Khyber Teaching Hospital, Peshawar, Pakistan)

EMAIL AND ORCID

ID: irida.dajti@gmail.com; 0000-0002-8449-6829

EV: enxhivrapi@gmail.com; 0000-0002-2767-5599

JK: jolamedicine16@gmail.com; 0000-0002-6893-477X

KSA: kirellossaid98@gmail.com; 0000-0003-0339-9339

FA: fatma.ahmed931977@gmail.com; 0000-0001-8456-1603

AES: aliaaeffat30@gmail.com; 0000-0002-3828-6038

SFA: shehabfathy134@gmail.com; 0000-0002-6964-247X

ZHH: zetairon@gmail.com; 0000-0001-6587-8517

MAN: mariam.16266094@med.aun.edu.eg; 0000-0002-7937-4229

ES: esraajamaleldeeb@gmail.com; 0000-0003-0290-5228

MS: moosa.shibani@gmail.com; 0000-0002-4424-2728

HI: Helmaismail997@gmail.com; 0000-0003-4329-9519

MAA: ameenzabibi@gmail.com; 0000-0003-4050-3051

BS: bishersawaf.94@gmail.com; 0000-0001-6751-619X

HAMO: hodaomran100@outlook.com; 0000-0003-1840-3599

CSR: chiristinesamuel@gmail.com; 0000-0002-7040-4268

MIAI: mohamedibrihem2012@gmail.com; 0000-0002-5216-7545

MSR: marinasamy25@gmail.com; 0000-0002-7691-2562

DMAH: arifulhaque58@gmail.com ; 0000-0003-4632-5153

RJA: dr.rifathjahan060@gmail.com; 0000-0003-0848-9456

SJN: elaine-1_ng@outlook.com; 0000-0001-5353-6499

JC: juwiechuah@live.com; 0000-0001-6992-3901

YLL: lim.95@hotmail.com; 0000-0001-6152-1959

YSJ: jeeys@hotmail.my

LCC: lichuinchong@gmail.com; 0000-0002-3574-1365

ICNR: giannisrocha@gmail.com; 0000-0002-8775-6876

KTC: kevincrispino16@gmail.com; 0000-0002-6505-2982

BY: nsbm2000@gmail.com; 0000-0001-6903-5146

AHMH: ahmed.hamed@students.kasralainy.edu.eg; 0000-0002-0843-532X

VU: venkatesh2007mbbs@gmail.com; 0000-0001-8059-9364

GJVM: moresoares11@gmail.com; 0000-0002-3531-0370

AVM: adrianaviola99@gmail.com; 0000-0001-8548-1592

RSA: aritonangrachel@gmail.com; 0000-0003-2702-2691

AI: imoto@nagasaki-u.ac.jp

DKK: kubotaa@yokohama-cu.ac.jp

KA: koji-aoki@chiba-u.jp

TTK: khanhtonthat08@gmail.com; 0000-0003-3462-4244

JMA: jeza1981@gmail.com; 0000-0002-1522-1880

MIMG: mohammed.jubar@gmail.com; 0000-0002-2873-3981

HFHK: dr.hemin@gmail.com; 0000-0002-8119-5246

RMH: rangen991@gmail.com; 0000-0001-5938-2224

SPD: sp.dumre@gmail.com; 0000-0002-4072-0745

PG: prgyawali@nhrc.gov.np;

MD: meghdhimal2@gmail.com; 0000-0001-7176-7821

KR: kamal_raj7@iom.edu.np; 0000-0002-3396-7440

PK: pallavik23@gmail.com

KA: adhkriti@gmail.com

RBD: renu.bdumre@gmail.com; 0000-0001-8039-0309

IU: irfanullahecp2@gmail.com; 0000-0003-1100-101X

FL: filipa-lucas@campus.ul.pt; 0000-0001-7651-7194

MP: kukushkinamd@gmail.com; 0000-0002-9564-6730

DP: pavlenkomd@gmail.com; 0000-0001-8761-9559

ATAQ: ahmadtqarawi@gmail.com; 0000-0001-7278-9889

JNPO: jn.pineda94@gmail.com; 0000-0002-0953-8742

JTOA: jtordonezaburto@gmail.com; 0000-0001-8705-4041

ASEL: andresestrella823@gmail.com; 0000-0003-1929-7548

BPGO: gom-olv@live.com.mx; 0000-0002-5905-8616

NK: nkoonrung@gmail.com; 0000-0003-4649-597X

CR: chidchanok.r@elearning.cmu.ac.th; 0000-0001-7927-1425

SN: surapon.nochaiwong@gmail.com; 0000-0003-1100-7171

PK: penkarnk@hotmail.com; 0000-0002-8160-5444

MN: k_mingkwan@hotmail.com; 0000-0003-4240-5367

IKT: irynakudlatska@gmail.com; 0000-0002-2700-1455

RP: tjroma1@gmail.com; 0000-0001-8070-5649

SI: somia.iqtadar@gmail.com; h0000-0002-8482-9355

UG: usman293eb@gmail.com

SUM: drsumumtaz@gmail.com

AAK: doctorasadalikhan@gmail.com; 0000-0001-8503-3036
