# Supplementary material for: Psychological Impacts and Post-Traumatic Stress Disorder among People under COVID-19 Quarantine and Isolation: A Global Survey
Source: Int J Environ Res Public Health. 2021 May 26;18(11):5719. doi: 10.3390/ijerph18115719 (PMC8199241; doi:10.3390/ijerph18115719)
Supplement: Supplementary file 1 [file ijerph-18-05719-s001.zip › ijerph-1180873-supplementary/supp/Supplementary-Table-S1.pdf]

Supplementary table S1. Country distribution of participants

Country	n (%)
Afghanistan	27 (2.9%)
Albania	8 (0.8%)
Algeria	6 (0.6%)
Australia	1 (0.1%)
Bahrain	1 (0.1%)
Bangladesh	87 (9.2%)
Bulgaria	1 (0.1%)
Canada	5 (0.5%)
Chile	3 (0.3%)
China	1 (0.1%)
Denmark	1 (0.1%)
Ecuador	45 (4.8%)
Egypt	34 (3.6%)
El Salvador	1 (0.1%)
France	31 (3.3%)
Germany	1 (0.1%)
Greece	5 (0.5%)
Holland	2 (0.2%)
Honduras	8 (0.8%)
Hungary	1 (0.1%)
India	56 (5.9%)
Indonesia	27 (2.9%)
Iraq	88 (9.3%)
Italy	1 (0.1%)
Japan	2 (0.2 %)
Jordan	3 (0.3 %)
Kazakhstan	1 (0.1%)
Korea	10 (1.1 %)
Kuwait	2 (0.2 %)
Lebanon	1 (0.1%)
Libya	32 (3.4 %)
Malaysia	8 (0.8 %)
Mexico	46 (4.9 %)
Myanmar	1 (0.1%)
Nepal	30 (3.2 %)

Netherlands	1 (0.1%)
New Zealand	1 (0.1%)
Nigeria	1 (0.1%)
Pakistan	46 (4.9%)
Palestine	34 (3.6%)
Philippines	34 (3.6 %)
Portugal	3 (0.3%)
Puerto Rico	1 (0.1%)
Qatar	8 (0.8%)
Romania	1 (0.1%)
Russia	1 (0.1%)
Saudi Arabia	14 (1.5%)
Sudan	15 (1.6%)
Syria	23 (2.4%)
Thailand	55 (5.8%)
Timor Leste	16 (1.7%)
Ukraine	13 (1.4%)
United Arab Emirates	4 (0.4%)
United Kingdom	12 (1.3%)
United States	16 (1.7%)
Vietnam	55 (5.8%)
Yemen	1 (0.1%)
Unknown	12 (1.3%)
**Total**	**944 (100%)**
